# Research on Creep Deformation of Dissimilar FSWed T-Joints Under Different Ultrasonic Vibration Modes: Experiment, Constitutive Model, and Simulation Verification

**DOI:** 10.3390/ma18102275

**Published:** 2025-05-14

**Authors:** Ti Ye, Yanjie Han, Duquan Zuo, Haoran Fu, Shilin Feng, Chong Gao, Wenya Li

**Affiliations:** 1 College of Aviation Engineering, Civil Aviation Flight University of China, 46, Nanchang Road, Guanghan 618307, China; yeti@cafuc.edu.cn (T.Y.); 18726537934@163.com (Y.H.); fuhaoran74@gmail.com (H.F.); fengshilin95@sohu.com (S.F.); 2 School of Engineering, University of Tokyo, 7-3-1 Hongo, Bunkyo, Tokyo 113-8656, Japan; 3 School of Materials Science and Engineering, Northwestern Polytechnical University, 127 West Youyi Road, Xi’an 710072, China; liwy@nwpu.edu.cn

**Keywords:** ultrasonic vibration, FSW, T-joints, creep deformation, constitutive model

## Abstract

This article presents experimental and numerical studies on the creep deformation of 7055-T6 Al and 2197-T8 Al-Li T-joints. Firstly, the optimal process parameters for creep tensile tests (CATs) are determined to be 155 °C, 130 MPa, and 8 h. Based on this, different modes of ultrasonic vibration are introduced. It is found that under the same amplitude, the creep limit of intermittent vibration is 64.7‰ to 97.2‰ higher than that of continuous vibration, and the tensile strength of the former specimens is significantly better than that of the latter. Further analysis reveals that during long-duration or high-amplitude vibrations, the joint material exhibits hardening effects, while short-duration, low-amplitude intermittent vibrations result in softening effects. When the amplitude is 12.53 μm, the material exhibits optimal comprehensive mechanical properties, with yield strengths, tensile strengths, and elongations of 402.1 MPa, 429.3 MPa, and 7.9%, respectively. Additionally, based on the mechanisms of superposition and acoustic softening effects, an improved creep aging constitutive model is established, which incorporates the creep process, stress superposition, and ultrasonic softening changes and is applied in ABAQUS. It is found that at an amplitude of 12.53 μm, the residual stress in the joint is more thoroughly eliminated and distributed more evenly, measuring 97.35 MPa. Moreover, the creep strain calculated using the above model in a finite element analysis shows a high degree of agreement with the experimental results, indicating that the proposed model can more accurately predict the creep deformation behavior of FSWed T-joints during the CAT process.

## 1. Introduction

With the development of fighter jets, large aircraft, and new transport planes in China, the third-generation, high-strength aluminum alloy 7055-T6 has received increasing attention in the aerospace field due to its high strength and high mass efficiency [1]. However, its high density leads to an increase in the overall weight of the large integral panel of fuselage. Friction stir welding (FSW), as an emerging solid-state joining technology [2], eliminates the need for riveting and reduces the overall structural weight [3]. However, Zuo et al. [4] found during the FSWed T-joint welding process that the heating of various regions of the T-joint was uneven. This resulted in a gradual decrease in the temperature of the weld nugget along the width of the T-joint, leading to complex changes in the microstructure and the uneven distribution of residual stresses. Farhang et al. [5] studied the effect of welding speed on residual stress using the drilling strain method and found that the longitudinal residual stress after friction stir welding decreases with increasing welding speed. Richter et al. [6] conducted a residual stress analysis on actual near-component samples using X-ray diffraction (XRD), and the results indicated that significant residual tensile stresses were generated at the surface of the weld in friction stir welding. Creep aging forming (CAF) is one of the key technologies for forming the large integral panels of aircraft. It aims to release residual stresses in the integral panel as much as possible through the simultaneous implementation of creep and artificial aging at a certain temperature, thereby achieving the formation and performance enhancement of the integral stiffened panel [7]. Compared to traditional forming methods, such as roll forming and shot peening, CAF specimens exhibit characteristics of less residual stress and a better forming performance [8,9], showing promising development prospects in the manufacture of large integral panels [10], especially for lightweight, large integral panels made by dissimilar alloy welding. By applying stress and temperature to the specimen over some time, the desired plastic deformation and good performance can be achieved.

Previous studies have mainly focused on the creep aging behavior of the same type of materials. Dong et al. [11] found through tensile tests that increasing the creep aging temperature can significantly enhance the creep strain of the AA 2050-T34 alloy and alter its creep behavior characteristics. Ma et al. [12] studied the creep behavior, mechanical properties, and microstructural evolution of Al-Cu-Li alloys under different stress levels, revealing that an increase in stress can refine the T1 precipitates and improve the yield strength of the creep aged samples. Zhang et al. [13] investigated the mechanical properties and microstructural evolution of Al-Cu-Li alloys during the CAF process, discovering that, compared to other artificial aging processes, CAF increased the number of T1 precipitates in the matrix, consequently enhancing the strength of the Al-Cu-Li alloy. However, after unloading, CAF specimens experience a significant rebound, which severely affects their forming accuracy. The introduction of ultrasonic vibration (UV) can effectively address these issues, not only reducing and homogenizing the residual stresses within the material but also lowering the forming force and improving the forming quality [14]. Yang and Li [15,16] utilized UV to reduce the residual stresses in diamond grinding tools and the deformation stresses in entropy alloys while improving their plastic strain. Liu et al. [17] performed ultrasonic vibration-assisted microtension tests on T2 copper foil, analyzing the fracture morphology to reveal the micro-mechanisms of the acoustic residual softening effect. Lyu et al. [18] discussed that rebound compensation and strength prediction are two key factors in the development of the CAF process. They also noted that a constitutive model for predicting the creep strain and mechanical properties of aluminum alloys during the CAF process has been developed to aid finite element (FE) process modeling. Zhang et al. [19] established a constitutive model that successfully predicted the tensile creep behavior of an Mg-8Gd-3Y-0.3Zr alloy under extrusion and T6 treatment. Li [20] proposed a unified constitutive model to predict the “multi-stage” creep behavior of an Al-Li-S4 alloy. To investigate the creep aging response of an 82.6% severely cold-rolled Al-Cu alloy under different loading directions, Chen et al. [21] established a mechanism-based constitutive model that accurately described the creep anisotropy of an Al-Cu alloy. Zuo et al. [22] established an improved Graham model that effectively characterized the creep characteristics of aluminum-alloy-stiffened panels under different stress levels during various creep stages. Li et al. [23] initially established and validated an FE model to simulate the asymmetric creep aging behavior of an aluminum–copper–lithium alloy (AA2050) during CAF. Under different stress levels, Peng et al. [24] studied the anisotropy of creep aging behavior in Al-Li alloys and established a constitutive model, finding good agreement between the predicted and the experimental values. However, many scholars have primarily focused on studies of the same material, with few reports on dissimilar materials. At present, most researchers focus on the creep aging of the same material, while there are few reports on the ultrasound-assisted creep aging behaviors of dissimilar materials.

In response, this paper focuses on the dissimilar FSWed T-joints, conducting CAT experiments to investigate the creep deformation and mechanical property changes of non-vibrated materials and to identify optimal creep process parameters. Under these conditions, ultrasonic vibration creep aging tensile (UVCAT) tests were performed. It was found that the appropriate amplitude could fully release the residual stress relaxation of the joint and improve the creep behavior of the FSW T-joint. Based on the mechanism of creep deformation, a constitutive model was established to describe the UVCAT process. Furthermore, the constitutive model was incorporated into the finite element software ABAQUS v2022, and the creep aging behavior of the T-joint was predicted using a creep subroutine to verify the accuracy and reliability of the finite element model, providing a reference for the high-quality manufacturing of lightweight integral panels.

## 2. Materials and Experiments

### 2.1. Materials

**Figure 1:**
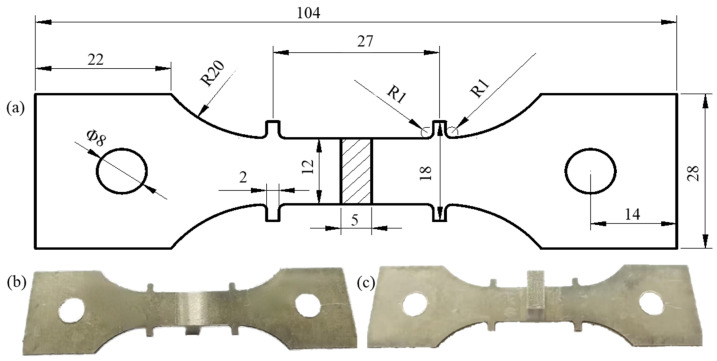
(**a**) The geometry of the T-joint specimen; (**b**) front and (**c**) back of views of the tensile specimens.

In this experiment, the pre-stretched 7055-T6 Al alloy [25] was used as the skin, with dimensions of 200 mm × 80 mm × 1.5 mm, and the 2197-T8 Al-Li alloy was used as the stiffener, with dimensions of 200 mm × 5 mm × 13 mm. The chemical composition and room temperature properties are shown in Table 1 and Table 2. According to the national standard [26], the T-stiffened plate was cut into aging tensile specimens along the fiber direction of the skin. The structure of the tensile specimens is shown in Figure 1a, and the physical images are shown in Figure 1b,c.

### 2.2. UVCAT Experiments

During the CAF process, temperature is the primary parameter affecting the creep or stress relaxation of joint materials. The creep temperature range for 7XXX series alloys typically falls between 120 °C and 200 °C [27]. Therefore, this experiment selected a temperature combination of (140 °C, 155 °C, 170 °C, and 180 °C). The CAT experiments are generally conducted under low-stress conditions. Hence, the chosen creep tensile load (*σ*) combinations were (130 MPa, 145 MPa, 160 MPa, and 175 MPa). Time is another parameter influencing material creep or stress relaxation, and the selected time combinations were (6 h, 8 h, 10 h, and 12 h). To quickly obtain optimal experimental points, a Taguchi orthogonal method [28] was employed to design the experimental scheme, as shown in Table 3.

In the self-made UVCAT instrument [29], a T-joint tensile specimen was installed. The ultrasonic power supply operated at 20 kHz, with a voltage of 220 ± 10 V and a power range of 800–2600 W. For this experiment, power levels of 1000 W, 1500 W, 2000 W, and 2500 W were selected to induce vibration in the specimen. The corresponding amplitudes for these power levels were 8.86 μm, 10.85 μm, 12.53 μm, and 14.01 μm, respectively. This study focuses on investigating the effects of different vibration modes on the creep deformation of T-joints, employing a comparative approach involving three conditions: no vibration, continuous vibration, and intermittent vibration, as shown in Table 4. The tensile tests were conducted on a GNCJ-100E micro-electronic creep tensile machine (Iron & Steel Research Institute Nake Testing Technology Co., Ltd., Beijing, China) at a constant strain rate of 1 mm/min. It is important to note that the flanges of the T-joint and the extensometer do not interfere with each other, being arranged in a 90-degree, staggered configuration.

## 3. Results and Discussion

### 3.1. Creep Aging Process Parameters

**Figure 2:**
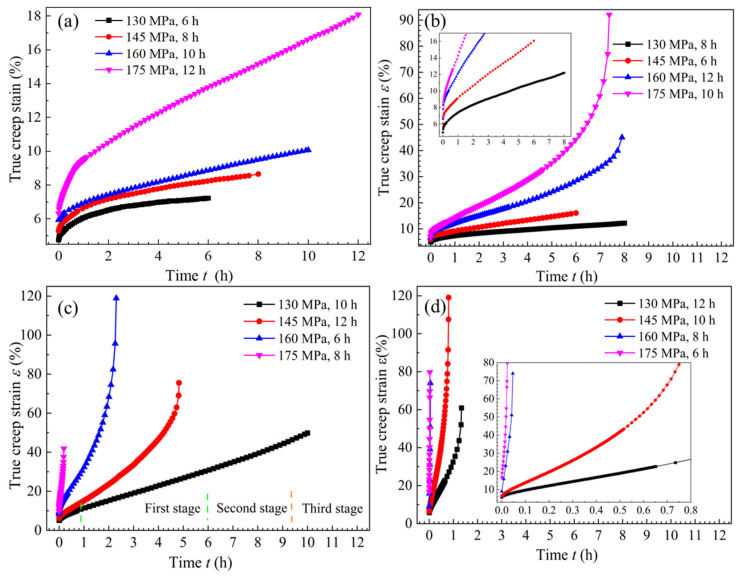
Real creep strain–time curve at (**a**) 140 °C, (**b**) 155 °C, (**c**) 170 °C, and (**d**) 185 °C.

Figure 2 shows the creep strain–time curves for the T-joint tensile specimens under non-vibration conditions, obtained according to Table 3. Observing Figure 2a–d, it can be seen that at a constant temperature, the creep strain rate of the tensile specimens increases with higher stress. However, when the loading stress is too high, the temperature is excessively elevated, or the aging time is prolonged, the premature fracture of the tensile specimens occurs, as indicated by A_2_B_3_C_4_, A_2_B_4_C_3_, A_3_B_2_C_4_, A_3_B_3_C_1_, A_3_B_4_C_2_, A_4_B_1_C_4_, A_4_B_2_C_3_, A_4_B_3_C_2_, and A_4_B_4_C_1_ in Figure 2. Since this study focuses on the creep deformation behavior and mechanical properties of the T-joint during UVCAT, the creep characteristics are only present in the first and second stages [30], without entering the creep acceleration stage. Therefore, this analysis will only explore the relationship within the first two stages of the creep characteristics, focusing specifically on the numbered parameters one to six in Table 3.

**Figure 3:**
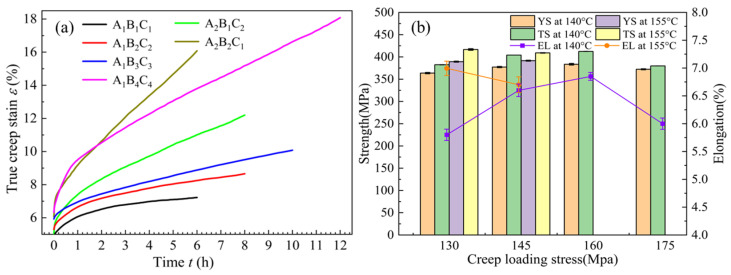
Relationship curves of (**a**) true creep strain–time and (**b**) mechanical property–loading stress.

Based on the creep strain versus time relationship curves under different creep temperature conditions, shown in Figure 2, and excluding curves that experienced premature fracture or entered the third creep stage, the creep curves at each stress level for 140 °C and 155 °C were obtained, as shown in Figure 3a. Observing Figure 3a, it can be seen that the steady-state creep time for plan A_2_B_1_C_2_ is shorter than that for A_1_B_3_C_3_, and the creep strain rate for the former is higher than that for the latter.

Figure 3b shows the mechanical performance curves corresponding to the T-joint under the conditions numbered one to six in Table 3. It can be observed that under a constant temperature of 140 °C, the yield strength (*YS*), tensile strength (*TS*), and elongation (*EL*) after fracture curves initially rise and then decline. The mechanical performance is optimal at a stress level of 160 MPa, with the *YS*, *TS*, and *EL* measuring 384.9 MPa, 411.7 MPa, and 6.8%, respectively. When the temperature increases to 155 °C, the *YS*, *TS*, and *EL* at a stress level of 130 MPa are 388.5 MPa, 417.8 MPa, and 7.1%, respectively, while at a stress level of 145 MPa, they are 390.7 MPa, 409.3 MPa, and 6.6%, respectively. Clearly, the *YS* of the material shows an increasing trend, but the growth is not significant, while *TS* and *EL* show a declining trend. In summary, at 155 °C and a stress level of 130 MPa, the *EL* is 0.3% greater than that at 140 °C and 160 MPa, indicating better plasticity. Additionally, the strength in the former case (*YS* = 388.5 MPa, *TS* = 417.8 MPa) is also greater by 3.6 MPa and 6.1 MPa, respectively, compared to the latter (*YS* = 384.9 MPa, *TS* = 411.7 MPa). Therefore, considering both the creep strain rate and mechanical performance, the performance of the specimen in plan A_2_B_1_C_2_ (155 °C, 130 MP, and 8 h) is superior to that of A_1_B_3_C_3_ (140 °C, 160 MPa, and 10 h), making its parameters the optimal creep aging process parameters for T-stiffened plates.

### 3.2. Creep Behavior Under Different Vibration Modes

**Figure 4:**
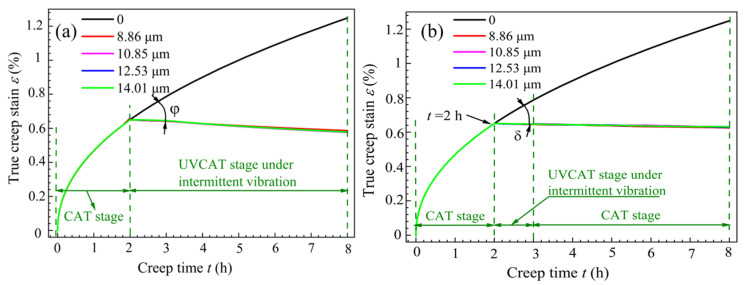
Creep–time curves under (**a**) continuous and (**b**) intermittent vibration.

Under the optimal creep aging process parameters, the effects of different vibration modes on the T-joint were studied. The creep strain–time curves for specimens under continuous and intermittent vibration during CATs are shown in Figure 4, where the angles between the amplitudes and the creep strain rates under no vibration are defined as *φ* and *δ*, respectively. From Figure 4a, it can be seen that during the first 2 h (the non-vibration period), the creep strain curves nearly coincide. As time progresses, the creep strain rate gradually decreases and stabilizes (entering the second creep stage), resulting in viscoplastic deformation. When the specimen is subjected to continuous vibration (2 h), regardless of the amplitude, there is a significant reduction in the creep strain rate, after which it enters a stable UVCAT process. During this process, the creep strain values also decrease as the creep time increases, which is attributed to the softening effect induced by vibration. At the end of the second creep stage, the steady-state creep limit under no vibration is 12.81‰, while the steady-state creep limits under amplitudes of 8.86 μm, 10.85 μm, 12.53 μm, and 14.01 μm are 5.87‰, 5.77‰, 5.78‰, and 5.79‰, respectively. This indicates that when the amplitude exceeds 10.85 μm, material hardening occurs.

Under intermittent vibration, the variation of the creep strain with time for the CAT specimens is shown in Figure 4b. Combined with Figure 4a, it can be seen that during the 2 h to 3 h period of applied vibration, the creep curves overlap, and the angle *δ* for the creep strain rate under intermittent vibration is smaller than the angle *φ* under continuous vibration. When vibration stops after 1 h, the creep strain in the material shows no significant change, but there still exists an angle *δ* concerning the non-vibration curve, and the creep curve under vibration is positioned below the non-vibration creep curve. Additionally, the steady-state creep limits for amplitudes of 8.86 μm, 10.85 μm, 12.53 μm, and 14.01 μm are 6.25‰, 6.31‰, 6.39‰, and 6.44‰, respectively. This indicates that intermittent vibration significantly affects the creep generation of CAT specimens, and the larger the amplitude, the higher the steady-state creep limit; however, the impact of angle *δ* on creep generation can be considered negligible. Compared to the steady-state creep limits under continuous vibration, it is found that at the same amplitude, the steady-state creep limits under intermittent vibration are greater by 64.7‰ to 97.2‰. In summary, under continuous vibration, when the amplitude exceeds 10.85 μm, material hardening occurs. Under intermittent vibration, the larger the amplitude, the smaller the angle of the creep strain rate, the larger the steady-state creep limit, and the greater the creep strain.

**Figure 5:**
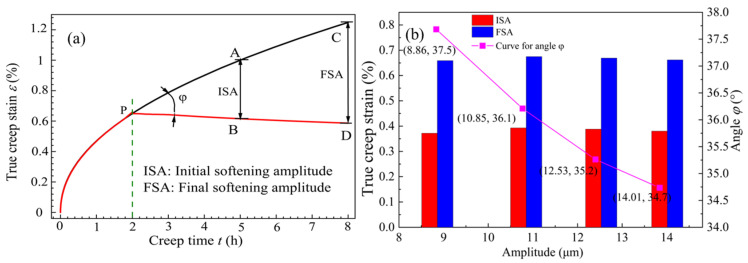
(**a**) ISA and FSA calibration plot and (**b**) ISA, FSA, and angle change curve under continuous vibration.

Based on the characteristics of the creep–time curves in Figure 4a, defining the difference between the creep value of the non-vibration curve at time t (point A) and the creep value of the vibrated curve at the moment the vibration begins (point B) as the initial softening amplitude (ISA). At the end of the vibration, the difference between the creep value of the non-vibration curve (point C) and the creep value of the vibrated curve (point D) is defined as the final softening amplitude (FSA). The calibration for ISA and FSA is shown in Figure 5a. The variation of the ISA (5 h) and the FSA (8 h) with increasing amplitude is illustrated in Figure 5b.

**Figure 6:**
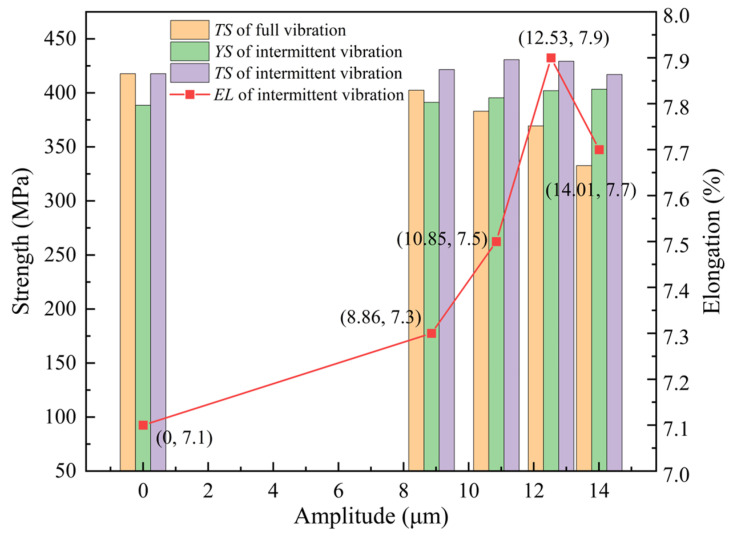
Mechanical properties of the T-joint under continuous and intermittent vibration.

From Figure 5b, it is evident that under continuous vibration, the magnitudes of the ISA and the FSA remain almost constant with changes in amplitude, indicating that the amplitude has little effect on the creep strain of the alloy during the aging process and can be considered negligible. Meanwhile, the *φ* value decreases as the amplitude increases, and when the amplitude exceeds 12.53 μm, the rate of decrease of *φ* becomes smaller, suggesting that significant amplitude in continuous vibration leads to material hardening. The comprehensive data analysis shows that an appropriate amplitude of continuous vibration results in the softening phenomenon of the alloy material during the creep process, as the externally applied UV hinders the creep strain of the tensile specimen, leading to a substantial decrease in *φ*. Since the creep strain rate decreases with increasing amplitude after vibration, there is no stress superposition effect in the alloy’s creep strain rate during the vibration softening process, which can be explained by the acoustic softening mechanism. To further clarify the above phenomenon, mechanical tensile experiments were conducted under different vibration conditions, with the tensile results for the T-joint shown in Figure 6.

From Figure 6, it can be observed that under continuous vibration, the *TS* of the T-joint decreases with increasing amplitude, and when the amplitude exceeds 12.53 μm, the rate of decrease in *TS* accelerates. This is due to large amplitude vibrations causing the material to undergo hardening, increasing creep resistance, reducing the conversion from elastic deformation to plastic deformation, and leading to the insufficient relaxation of the material, which prevents adequate stress release and results in an uneven distribution of internal stress, thereby decreasing the material strength. Under intermittent vibration, the *YS* of the material increases with increasing amplitude, and the *TS* shows an increasing trend from 0 to 10.85 μm and then decreases from 10.85 μm to 14.01 μm. The *EL* initially increases and then decreases with increasing amplitude, rapidly increasing when the amplitude exceeds 10.85 μm and quickly decreasing beyond 12.53 μm. This is because, during short periods of intermittent vibration, low amplitudes exhibit a softening effect, reducing the creep stress resistance of materials and allowing the residual stress to be fully released. However, as the amplitude increases, the hardening effect gradually dominates, reducing the mechanical properties of the T-joint. The graph also shows that, compared to the same amplitude in continuous vibration, the *TS* of the T-joint under intermittent vibration is significantly better. Additionally, under intermittent vibration, the combination of mechanical properties is optimal at an amplitude of 12.53 μm, with the *YS*, *TS*, and *EL* being 402.1 MPa, 429.3 MPa, and 7.9%, respectively.

### 3.3. Constitutive Model

The change in the stress softening of the T-joint during the UVCAT process primarily comes from the stress superposition effect and the acoustic softening effect. The variation in creep softening is mainly attributed to the acoustic softening effect, both of which occur during the viscoplastic process of the second stage of creep. Since the time during the creep loading phase is relatively short, and the externally applied stress is much lower than the yield strength of materials, it is assumed that during the loading phase the material only experiences elastic deformation, while its plastic deformation is negligible. Therefore, the entire deformation process of the tensile specimen during the UVCAT process can be simplified into two stages of strain: stage one is elastic strain, which is the strain *ε*_e_ resulting from elastic loading, and stage two is viscoplastic strain, which is the sum of the creep strain *ε*_c_ and the strain *ε*_u_ resulting from vibration softening. Thus, the stress–strain relationship throughout the UVCAT process can be expressed as follows:

(1) $$\left\{\begin{array}{c} \varepsilon ={\;\varepsilon }_{e}+\varepsilon_{c}+\varepsilon_{u}\\ \sigma ={\sigma_{s}}^{0}+{\;\sigma }_{c}+\sigma_{supeerposition}-∆\sigma_{sofening} \end{array}\right.$$

In the formula, *ε_e_* = *σ_s_*^0^/*E*, *E* is the elastic modulus, ${\sigma_{s}}^{0}$ is the elastic stress, $\sigma_{c}$ is the creep stress, σ_supeposition_ represents the stress superposition effect, and σ_softening_ represents the acoustic softening effect.

During the CAT process, the creep of the material and the UV occur simultaneously and interact with each other. To accurately describe the relationship between the softening (amplitude) and the creep strain of materials, a state variable *Φ* is introduced to represent the variation in material amplitude, which is defined based on the relationship between *H* and H* [31]. That is,

(2) $$\frac{d\Phi }{\mathrm{dt}}=C_{\Phi }{{\dot{\varepsilon }}_{e}}^{n_{2}}{\left(\Phi^{*}-\Phi \right)}^{\;n_{3}}$$

where *C*_Φ_, *n*_2_, *n*_3_, and Φ* are constants. Φ* represents the saturation level of *Φ*. As *Φ* approaches Φ*, the change in its value can describe the variation in energy that affects material softening during the amplitude process. Thus, the effect of input energy on material softening can be expressed as follows:

(3) $$\varepsilon_{u}={C_{A}(1+\frac{\Phi }{\Phi^{*}})}^{n_{4}}$$

In the equations above, *C*_A_ and *n*_4_ are constants, and $\varepsilon_{u}$ represents the softening strain. Based on the dislocation damping theory [32], this is the material’s formula for dislocation damping energy. Therefore, the relationship expression between amplitude and vibration energy, Q, under the ultrasonic softening effect is derived as follows:

(4) $$\left\{\begin{array}{c} Q=1/{\left(\alpha \rho L\right)}^{2}=\rho w^{2}A^{2}\\ {∆\sigma_{\mathrm{sofening}}=-2m_{0}\sigma (Q/\sigma )}^{m_{1}} \end{array}\right.$$

In the equation, α is a constant; L and ρ represent the linear density and distribution density of mobile dislocations, respectively; *A* is the amplitude; ω is the angular frequency; and m_0_ and m_1_ are constants.

*K* is a linear strengthening function related to the amplitude A. The value of *K* (A) corresponds to the ratio of the linear strengthening coefficients of the alloy in the viscoplastic stage under vibration to those under static conditions after vibration is applied. It can be expressed as follows:

(5) $$K(A)=\eta_{1}/(\eta_{1}+{\;\eta }_{2})$$

In the equation, η_1_ and η_2_ represent the viscous strain coefficients for the elastic and plastic components, respectively. Considering the combined interaction mechanisms of subsequent yielding effects, strain rate variations, strain evolution, and elastic strain [33], the dynamic stress function based on viscoelastic–plastic theory can be comprehensively derived as follows:

(6) $$\sigma \;=\frac{\eta_{1}\eta_{2}}{\eta_{1}+\eta_{2}}(\dot{\varepsilon }+\frac{E\varepsilon_{e}}{\eta_{1}}+\frac{\sigma_{s}+K(\varepsilon -\varepsilon_{0}-\varepsilon_{e})}{\eta_{2}})$$

In the equation, σ and ε are the stress and strain, respectively; $\dot{\varepsilon }$ is the strain rate; *E* is the elastic modulus; ε_e_ and σ_s_ denote the elastic strain and material yield strength, respectively; and ε_0_ represents the quasi-static strain. By substituting Equation (5) into Formula (6) and organizing and simplifying it, we can obtain the stress–strain function of the alloy under the stress superposition effect, as follows:

(7) $$\sigma \;=BK(A)\varepsilon \;+K(A)\dot{\varepsilon }+\sigma_{S}$$

**Figure 7:**
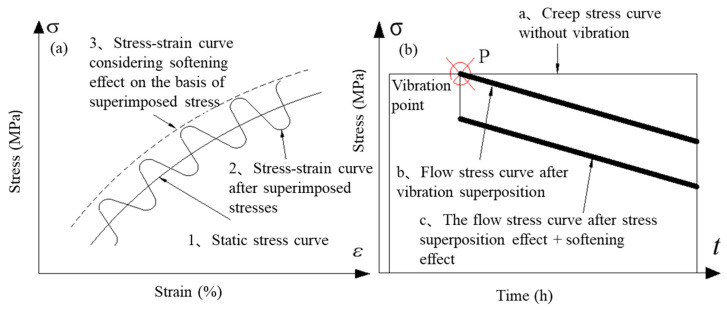
Superposition of (**a**) stress–strain and (**b**) creep stress–time curves of materials under UV.

Figure 7 shows the superimposed state diagram of the stress–strain and creep stress–time curves of the material under UV. Since the creep process occurs in the viscoplastic stage, the T-joint material has already started to yield, but the amplitude rod of the vibrator remains in a state of elastic deformation. Therefore, the stress superposition effect is still applicable to the amplitude rod. The load (or displacement) at the connection point between the amplitude rod and the fixture head is a vector summation of the two.

Figure 7a is an enlarged state diagram of the curves. As shown, the oscillation of stress and the yield stress of the material during creep are superimposed in real-time, and with the effect of acoustic softening, the stress–strain curve of the materials is shifted (see dashed line 3 in Figure 7a). Figure 7b shows the variation state of creep stress under stress superposition. By combining the above models of stress superposition and acoustic softening, it can be concluded that the decrease in the flow stress of aluminum induced by UV in the UVCAT is the algebraic sum of the stress superposition and acoustic softening effects (*σ_superposition_* − Δ*σ*_softening_). Therefore, the creep stress generated in the viscoplastic phase of the UVCAT can be expressed as follows:

(8) $$\left\{\begin{array}{c} \sigma_{e}=\sigma -\sigma s^{0}-∆\sigma ,\;\;\sigma \;\geq \;{\sigma_{s}}^{0}-∆\sigma \\ ∆\sigma =\sigma_{supeerposition}-∆\sigma_{sofening} \end{array}\right.$$

Here, *σ*_superposition_ represents the stress superposition effect, while Δ*σ*_softening_ represents the acoustic softening effect.

**Figure 8:**
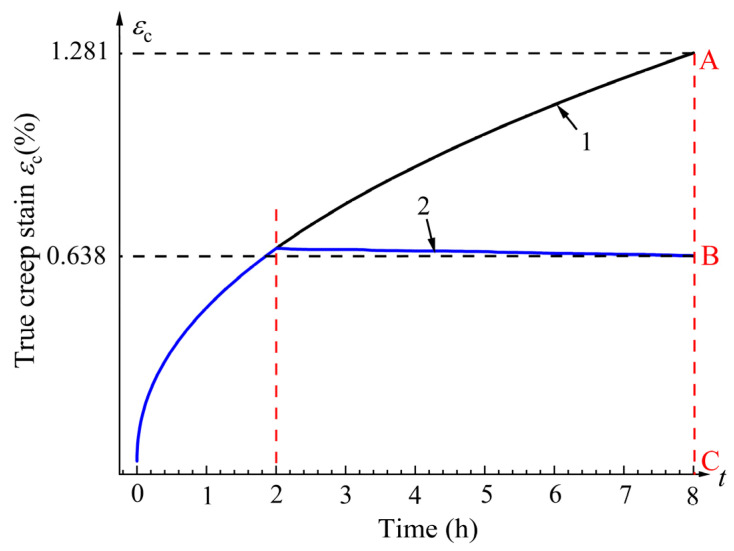
Creep–time curves of T-joints with/without ultrasonic intermittent vibration. (1) Curve 1 is a creep curve without vibration. (2) Curve 2 shows the creep curve with an amplitude of 12.53 μm.

Due to the influence of softening effects during material forming, the creep stress state (or creep strain) and yield strength in the viscoplastic stage will decrease by Δ*σ*, while the amount of stress release will increase by Δ*σ*. Additionally, the superimposed stress effect will lead to a decrease in the linear strengthening coefficient of materials. These factors indicate that the creep constitutive relationship of the material has been altered under UV. Figure 8 shows the creep curves at amplitudes of 0 μm and 12.53 μm from Figure 4a. As can be seen from the figure, UV reduces the steady-state creep limit of the material and lowers the creep power law, resulting in a greater transformation of elastic deformation into plastic deformation. Although the stress release generated by creep decreases, the softening effect caused by UV allows for a more complete plastic flow of the material, leading to a greater reduction of residual stress and more thorough overall stress release. This has significant implications for improving material forming precision in industrial applications. Furthermore, the creep aging effect provides the residual stress of materials with more time to oscillate and dissipate in conjunction with the stress induced by vibration. The UV in the CAT is essentially an energy output process, and it can be inferred that UV alters the creep constitutive relationship of the tensile specimen, causing creep tensile curve 1 (creep limit 12.81‰) in Figure 8 to decline to curve 2 (creep limit 6.38‰). The creep curve of the T-joint is attributed to power law hardening, expressed as follows:

(9) $$\sigma =\alpha \varepsilon^{k_{0}}$$

where *α* is a constant, and *k*_0_ is the creep power law hardening index, with *σ* and *ε* representing stress and strain, respectively.

If UV affects *k*_0_ in Equation (9), we assume the constitutive relationship of the material under UV to be as follows:

(10) $$\sigma =\alpha \varepsilon^{k_{1}}$$

where *k*_1_ is the vibration power law hardening index. To describe the relationship of the creep variation between intermittent vibration and no vibration, vertical lines are drawn at the creep limits of curves 1 and 2 (*t* = 8 h) to intersect at points A and B. Measurements reveal that the length ratio AC:AB is 1:0.48. Similarly, for other points along curves 1 and 2, vertical lines are drawn to measure lengths, indicating a ratio of 1:0.48e^(8−*t*)tan*δ*^. This suggests that the creep strain under UV is 0.48e^(8−*t*)tan*δ*^ times that under the non-vibration condition. Therefore, Equations (9) and (10) have the following relationship:

(11) $$\alpha \varepsilon^{k_{1}}=0.48e^{\left(8-t\right)\tan\varphi }\alpha \varepsilon^{k_{0}}$$

By taking the logarithm, we can derive that *k*_1_ = *k*_0_ + log0.48 + (8 − *t*)tan*δ* = *k*_2_ + (8 − *t*)tan*δ*. This relationship indicates that the power law hardening index *k*_1_ under vibration is not a constant but rather a parameter related to the strain *ε*. In this study, since the creep characteristics in the UVCAT only exhibit the first and second stages and do not enter the creep acceleration stage, we omit the parameters related to creep damage from the constitutive model in reference [31]. Compared to traditional simple models, the modified creep constitutive equations can better describe the variation of creep strain during the CAT process of the material, especially the creep characteristics of the second stage. The specific equations are as follows:

(12) $$\left\{\begin{array}{c} {\dot{\varepsilon }}_{c}=C_{1}\;sin\;h[C_{2}\;(\sigma -\sigma_{0}){(1-H)}^{n_{0}}]\\ \dot{H}=h\;/\;\sigma^{n_{1}}(1-H\;/{\;H}^{*}){\dot{\varepsilon }}_{c} \end{array}\right.$$

where *C*_1_, *C*_2_, H*, *n*_0_, *n*_1_, and *h* are material constants, ${\dot{\varepsilon }}_{c}$ is the creep strain rate of the material, *σ* is the applied stress, σ_0_ is the internal stress considering impediments to dislocation movement, and *H* is a control variable affecting the course of the creep curve, reflecting how material hardening influences the creep rate changes during the first creep stage. Given the significant impact of UV on the creep strain rate of materials, the following correction is made to Equation (12) [34]:

(13) $${\dot{\varepsilon }}_{c}=C_{1}\sin\;h\;[C_{2}(\sigma -\sigma_{0}){(1-H)}^{n_{0}}\;{(1+A/A^{*})}^{K_{2}+(t_{1}-t_{2})\;\tan\varphi }]$$

By combining Equations (1)–(13), obtaining an improved creep aging constitutive relationship that incorporates the creep process, stress superimposition, and ultrasonic softening variations in the UVCAT can be achieved as follows:

(14) $$\left\{\begin{array}{c} {\dot{\varepsilon }}_{c}=C_{1}sin\;h\;[C_{2}(\sigma -\sigma_{0}){(1-H)}^{n_{0}}\;{(1+A/A^{*})}^{K_{2\;}+(t_{1}-t_{2})\;\tan\varphi }]\\ \dot{H}=h\;/\;\sigma^{n_{1}}(1-H\;/{\;H}^{*}){\dot{\varepsilon }}_{c}\\ \dot{\Phi }=C_{\Phi }{{\dot{\varepsilon }}_{C}}^{n_{2}}{\left(\Phi^{*}-\Phi \right)}^{\;n_{3}}\\ \varepsilon_{u}={C_{A}(1+\frac{\Phi }{\Phi^{*}})}^{n_{4}}\\ Q=\rho w^{2}A^{2}\\ {∆\sigma_{sofening}=-2m_{0}\sigma (Q/\sigma )}^{\;m_{1}}\\ \;\sigma_{superposition}=K(A)B\varepsilon +K(A)\dot{\varepsilon }+\sigma_{S}\\ \sigma =\sigma_{c}+\sigma_{superposition}-∆\sigma_{sofening\;},\;\sigma \;\geq {{\;\sigma }_{S}}^{0}-∆\sigma \\ \varepsilon ={\;\varepsilon }_{e}+\varepsilon_{c}+\varepsilon_{u} \end{array}\right.$$

In the equation, *C*_1_, C_2_, *h*, *σ*_0_, H*, *n*_0_ ~ *n*_4_, *m*_0_ ~ *m*_1_, *C*_Φ_, *C*_A_, Φ*, *B*, *k*_0_, and *k*_2_ are all material parameters. *A* is the real-time amplitude corresponding to the current UV, A* is the maximum value of the current vibration amplitude, *t*_1_ is the total creep time, *t*_2_ is the time during which vibration is applied, and *δ* is the angle between the slope of the creep curve at the point of vibration application and the softening curve. Furthermore, ${\dot{\varepsilon }}_{u}$ and $\dot{H}$ describe the variation in the creep strain rate during the UVCAT process; $\dot{\Phi },$ $\varepsilon_{u}$, and *Q* are intermediate relationships related to amplitude, energy, and material softening; and *σ* and *ε* describe the stress–strain relationship of the material under the stress superimposition and the softening effects during the UVCAT process.

Due to the numerous material constants in the constitutive equation, the Runge–Kutta method is employed to accurately characterize the creep behavior of the material during the creep aging tensile process under UV. These material constants are treated as optimization variables, with the creep strain error between the experimental data and the fitting points at corresponding locations as the optimization objective function. The fourth-order Runge–Kutta method and genetic algorithm (GA) [35] were utilized to perform integration calculations and optimize the constitutive relationship expressed in Equation (14), resulting in the determination of the material parameters in the creep aging constitutive equation affected by intermittent vibration, as shown in Table 5.

**Figure 9:**
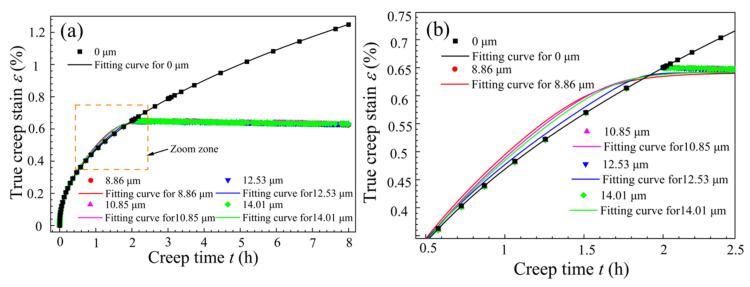
The creep strain fitting results for T-joint materials: (**a**) the creep fitting curve; (**b**) an enlarged view of the orange dashed box in figure (**a**).

Based on the experimental data from Figure 4a and the established constitutive model, the curve fitting the real creep strain as a function of time was obtained, as shown in Figure 9.

From Figure 9a, it can be seen that under non-vibration conditions, the creep strain of the T-joint gradually increases over time. When it enters the second creep stage, the creep rate becomes constant, and the creep strain increases uniformly. For intermittent vibration, before the application of vibration, the vibration curve coincides with the creep curve under no vibration and exhibits the same trend. After applying UV, the creep strain rate of the material experiences an instantaneous drop *δ*, and then the creep strain rate approximates a straight line, with the creep value decreasing uniformly and slowly. The overall trend of the fitted curve matches well with the experimental data points, with the maximum fitting error for the creep strain occurring just before the application of vibration (see the orange dashed box in Figure 9a), amounting to 4.2%, which is less than 5%, indicating a high fitting accuracy. This demonstrates that the established creep constitutive model can effectively describe the creep behavior of the T-joint material at 155 °C and 130 MPa. From Figure 9b, it can be observed that the fitted creep curve with an amplitude of 12.53 μm is closer to the creep fitted curve under no vibration, with its minimum error being 1.6%. Thus, it can be further inferred that the processing parameter of 12.53 μm can more accurately describe the creep behavior during the creep aging tensile process of the T-joint.

## 4. Finite Element Validation

**Figure 10:**
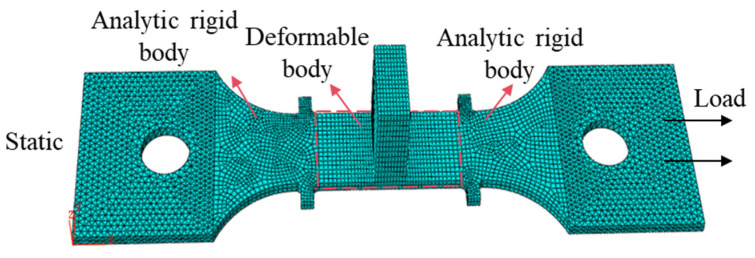
A finite element model with the loading conditions of the T-joint.

Although the established constitutive equation can accurately predict the creep behavior during the creep aging tensile process of the T-joint, it is still necessary to further develop it within finite element software to verify its reliability. Therefore, a finite element model consistent with the geometric dimensions of the T-joint specimen in the experiments was established using ABAQUS software, divided into three segments: left, middle, and right. The left and right segments of the gauge length (red dashed boxes) are set as analytical rigid bodies, while the middle segment is set as a deformable body, as illustrated in Figure 10. In the right section, a reference point is set for the rigid body to define the external forces and boundary conditions. Material properties are assigned, including density, Poisson’s ratio, elastic modulus, and creep aging parameters. The improved creep constitutive model is incorporated into the model as a subroutine, and the application of the ultrasonic vibration signal is realized using the * Amplitude/Periodic type. Periodic motion is applied to the right end of the specimen according to the amplitude curve. The established model contains 23,484 nodes and 16,524 elements, of which there are 16,308 C3D8R linear hexahedral elements and 214 C3D6 linear wedge elements. The previously established creep aging constitutive model (Equation (14)) and the corresponding material parameters from Table 5 are seamlessly embedded into the ABAQUS model via a creep subroutine. The material density is set to 2.7 g/cm^3^, the elastic modulus to 69 GPa, the ratio of Poisson to 0.3, and the other parameters are set to the same values used in the experiments are 130 MPa, 155 °C, and 8 h, respectively.

The simulation of the UVCAT experiment is conducted in multiple steps:

(1) Loading phase: the left end of the specimen is fully constrained and stationary, the right end is linearly loaded in the X direction, nonlinear analysis is turned off, and the conventional tensile time without vibration is 1 s.
(2) CAT phase: the left end of the specimen remains stationary, the right end undergoes constant loading in the X direction, nonlinear analysis is enabled, and the time for the non-vibration CAT is set to 2 h.
(3) UVCAT phase: the left end of the specimen remains stationary, while both a sine displacement function and a constant load are applied at the right end, with a vibration duration of 1 h.
(4) CAT phase: the left end of the specimen remains stationary, and the right end continues to receive constant load in the X direction, with a non-vibration CAT applied for 5 h.
(5) Unloading phase: the left end of the specimen remains stationary, the right end is linearly unloaded in the X direction, the temperature load is reduced to 25 °C, and the time for the non-vibration CAT is 1 s.

**Figure 11:**
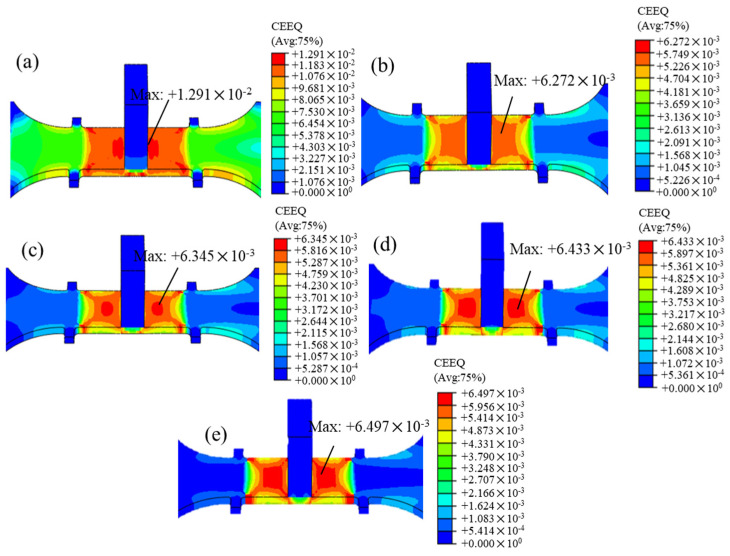
Equivalent creep strain distribution after aging for 8 h with no and different amplitudes (1 h excitation): (**a**) 0 μm, (**b**) 8.86 μm, (**c**) 10.85 μm, (**d**) 12.53 μm, and (**e**) 14.01 μm.

The distribution of the equivalent creep strain after the T-joint material undergoes non-vibration CAT and intermittent vibration UVCAT processing is shown in Figure 11. It can be observed that under the creep load, the maximum creep strain of the material at the end of the creep period is 1.291 × 10⁻^2^ (0 μm). When the amplitude is 8.86 μm, the maximum creep strain of the material decreases to 6.272 × 10⁻^3^. However, as the amplitude increases, the maximum creep strain similarly increases. Additionally, it can be seen from the figure that the maximum creep strain occurs in the gauge length segment of the specimen, particularly near the stiffener. Under no vibration, the distribution of creep values in the gauge length segment is relatively uniform, showing a larger amount of creep generation. However, after applying vibration, the creep strain distribution in the gauge length segment becomes uneven with areas showing more creep distributed in a concave trapezoidal shape. A comparison of the simulated and the experimental results shows that the simulated creep values are consistently greater than the experimental results, which may stem from the simplification of the weld zone in the finite element geometric model. Nevertheless, when there is no vibration, the maximum error between the two is only 0.1‰, indicating a high level of credibility for the established creep constitutive model.

**Figure 12:**
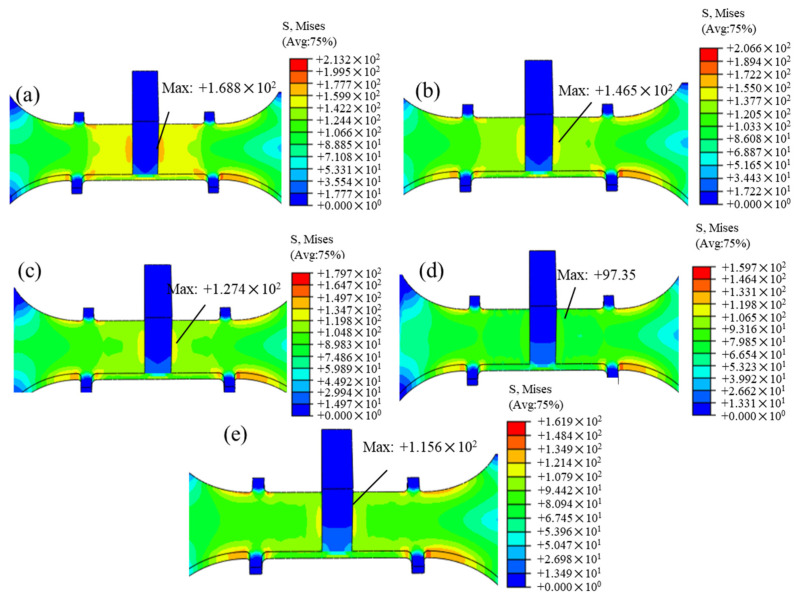
Residual stress changes after aging for 8 h under no and different amplitudes (1 h excitation): (**a**) 0 μm, (**b**) 8.86 μm, (**c**) 10.85 μm, (**d**) 12.53 μm, and (**e**) 14.01 μm.

Figure 12 illustrates the changes in the residual stress of the T-joint material after 8 h of the creep aging treatment under different amplitude conditions. Figure 12a shows the residual stress distribution after creep aging tensile under no vibration, where the maximum residual stress is located near the stiffener, at 168.8 MPa, with the research area appearing in orange and a stress variation range of 159.9 to 177.7 MPa. After applying UV, under the dual effects of stress superposition and ultrasonic softening, the residual stress in the research area gradually diminishes. At an amplitude of 12.53 μm, the stress release is most thorough, with the residual stress in the research area showing a uniform distribution in green, and the maximum residual stress being 97.35 MPa. However, when the amplitude is increased to 14.01 μm, the maximum residual stress increases and the stress distribution exhibits unevenness. This is likely due to excessive amplitude and high input energy causing an uneven stress distribution within the material, which aligns with the conclusions drawn from previous experiments.

The simulation results indicate that although the CAT specimen without vibration generates a larger amount of creep strain, the residual stress in the material is significantly higher and exhibits a highly uneven distribution after aging. This explains why components formed using traditional CAF technology experience significant rebound and uncontrolled forming precision and can only achieve 50–70% of the forming effect [36]. An appropriate amplitude during the auxiliary CAT process helps reduce the generation of creep strain due to ultrasonic softening, while the dual effects of stress superposition and softening facilitate more thorough stress relief, resulting in a more uniform distribution of residual stress after aging.

**Figure 13:**
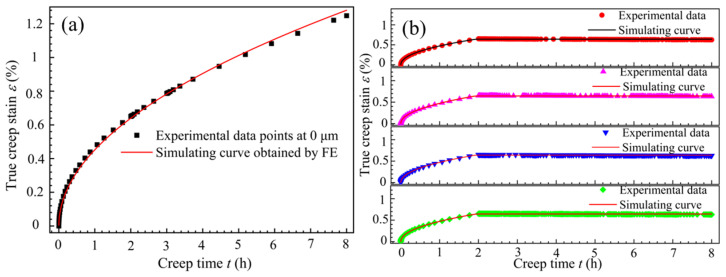
Comparison of creep strain experimental data and finite element simulation results at different amplitudes: (**a**) 0 μm, (**b**) 8.86 μm, 10.85 μm, 12.53 μm, and 14.01 μm.

The comparison of the creep strain experimental data at different amplitudes with finite element simulation results is shown in Figure 13. It can be observed that, whether under no vibration or with vibration, the creep strain rate before vibration exhibits significant variation. After vibration, the creep strain rate approaches a constant value, and the simulation results of the creep curve before vibration correlate better with the experimental data.

Additionally, the fitted curves consistently lie above the experimental data points, with the maximum creep error being 3.8 × 10⁻^4^. This phenomenon may originate from the simplification of the welding zone when establishing the finite element model. To ensure computational convergence, the concave surface formed by the downward pressure of the stirring tool was simplified to a plane. This simplification leads to a greater effective thickness of the welding joint compared to the experimental data, resulting in a higher generation of creep. Overall, the calculated creep values from the finite element analysis show a high degree of agreement with the experimental data, thereby validating the reliability and accuracy of the derived creep constitutive model from another perspective. This model effectively reflects the creep behavior of the T-joint material during the UVCAT process. Moreover, it provides a theoretical foundation for the identification of large, integral, stiffened plates, which is of significant importance for the manufacturing of low-density integral wall structures.

## 5. Conclusions

This paper presents an experimental and numerical study on the creep deformation behavior of dissimilar Al/Al-Li T-joints. Initially, CAT experiments were performed to determine the optimal creep processing parameters. Under these conditions, UVCAT experiments with different vibration methods were conducted to analyze the creep deformation of materials, creep strain rates, and mechanical properties. Furthermore, an improved creep aging constitutive model based on physical mechanisms was established for numerical investigation, and the reliability and the accuracy of the finite element model were verified. The following conclusions can be drawn:

(1) Under non-vibration conditions during the CAT experiments, the optimal creep processing parameters for the T-joint were 155 °C, 130 MPa, and 8 h.
(2) The UVCAT experiments revealed that at the same amplitude, the creep limit for intermittent vibration was 64.7‰ to 97.2‰ larger than that for continuous vibration. Furthermore, compared to the TS of the CAT specimen under continuous vibration, the specimens under intermittent vibration significantly outperformed them. It was further found that under prolonged or large amplitude vibrations, the material exhibits a hardening effect. Whereas, under short-duration, low-amplitude, intermittent vibrations, the joint material showed a softening effect with sufficient release of residual stress. The optimal composite mechanical properties of the material occur at an amplitude of 12.53 μm, with the YS, TS, and EL being 402.1 MPa, 429.3 MPa, and 7.9%, respectively.
(3) Based on the analysis of the UVCAT experiments, a creep aging constitutive model incorporating the mechanisms of superposition and acoustic softening effects was established. The fit to the experimental results yielded an error of 4.2%, which is less than 5%, satisfying engineering practical needs and confirming the accuracy of the model.
(4) The simulation results indicated that an amplitude of 12.53 μm, while reducing the generation of creep strain in the CAT specimens, led to a more thorough elimination of residual stress, with a more uniform distribution at 97.35 MPa. Moreover, the finite element calculated creep strain matched the experimental results well, with a maximum error of 3.8 × 10⁻^4^, further demonstrating that the creep constitutive model can effectively predict the creep behavior of T-joint materials during the UVCAT process.

## Figures and Tables

**Table 1:** Chemical composition of Al 7055-T61 and Al-Li 2197-T8 alloys (mass fraction, %).

7055-T61 alloy	Zn	Mg	Cu	Zr	Fe	Mn	Si	Ti	Cr	Al
	7.9	2.1	2.3	0.05	0.15	0.05	0.1	0.06	0.001	Bal.
2197-T8 alloy	Li	Cu	Ag	Mn	Mg	Zr	Fe	Si	Zn	Al
	1.5	2.88	0.36	0.35	0.24	0.09	0.05	0.04	0.006	Bal.

**Table 2:** Room temperature properties of 7055-T61 and 2197-T8 alloys.

Materials	Transverse Tensile Strength/MPa	Longitudinal Tensile Strength/MPa	Elongation/%	Melting Point/°C
7055-T6	600	540	13	590
2197-T8	570	530	6	560

**Table 3:** Creep aging tensile experiment scheme.

No.	Creep Temperature/°C	Loading Stress/MPa	Creep Time/h	Combination
1	140	130	6	A_1_B_1_C_1_
2	140	145	8	A_1_B_2_C_2_
3	140	160	10	A_1_B_3_C_3_
4	140	175	12	A_1_B_4_C_4_
5	155	130	8	A_2_B_1_C_2_
6	155	145	6	A_2_B_2_C_1_
7	155	160	12	A_2_B_3_C_4_
8	155	175	10	A_2_B_4_C_3_
9	170	130	10	A_3_B_1_C_3_
10	170	145	12	A_3_B_2_C_4_
11	170	160	6	A_3_B_3_C_1_
12	170	175	8	A_3_B_4_C_2_
13	185	130	12	A_4_B_1_C_4_
14	185	145	10	A_4_B_2_C_3_
15	185	160	8	A_4_B_3_C_2_
16	185	175	6	A_4_B_4_C_1_

**Table 4:** Process parameters under different vibration conditions.

No.	Frequency/KHz	Amplitude/µm	Tension Rate/mm·min^−1^	Vibration Application Method
1	0	0	1	No
2	20	8.86	1	Continuous vibration of 2 h
3	20	10.85	1	
4	20	12.53	1	
5	20	14.01	1	
6	20	8.86	1	2~3 h intermittent vibration
7	20	10.85	1	
8	20	12.53	1	
9	20	14.01	1	

**Table 5:** Material parameters of improved creep aging constitutive equation.

Parameter of Equation	Parameter Value	Parameter of Equation	Parameter Value
*C*_1_/h^−1^	3.166 × 10^−10^	*m*_0_	2.650 × 10^−11^
*C*_2_/MPa^−1^	8.742 × 10^−2^	*m*_1_	2.307
*h*/MPa	1.483 × 10^5^	*C*_Φ_	0.132
*σ*_0_/MPa	256.473	*C*_A_	18.304
H*	0.2716	Φ*	1.526 × 10^−4^
*n*_0_	3.0175	*B*	0.0537
*n*_1_	0.0327	*K*(A)	0.6835
*n*_2_	2.1068	*k*_0_	2.6783
*n*_3_	1.3685	*k*_2_	2.3595
*n*_4_	−23.671		

## Data Availability

The original contributions presented in this study are included in the article. Further inquiries can be directed to the corresponding authors.
